# From the nucleation of wiggling Au nanostructures to the dome-shaped Au droplets on GaAs (111)A, (110), (100), and (111)B

**DOI:** 10.1186/1556-276X-9-113

**Published:** 2014-03-12

**Authors:** Ming-Yu Li, Mao Sui, Eun-Soo Kim, Jihoon Lee

**Affiliations:** 1 College of Electronics and Information, Kwangwoon University, Nowon-gu, Seoul 139-701, South Korea; 2 Institute of Nanoscale Science and Engineering, University of Arkansas, Fayetteville, AR 72701, USA

**Keywords:** Self-assembled Au droplets, Annealing temperature, Various surface indices, Nucleation

## Abstract

In this paper, the systematic evolution process of self-assembled Au droplets is successfully demonstrated on GaAs (111)A, (110), (100), and (111)B. On various GaAs substrates, self-assembled Au clusters begin to nucleate at around 300°C, and then, they develop into wiggly Au nanostructures at 350°C. Between 400°C and 550°C, the self-assembled dome-shaped Au droplets with fine uniformity are fabricated with various sizes and densities based on the Volmer-Weber growth mode. Depending on the annealing temperature, the size including the average height and lateral diameter and the density of Au droplets show the opposite trend of increased size with correspondingly decreased density as a function of the annealing temperature due to the difference in the diffusion length of adatoms at varied activation energy. Under an identical growth condition, depending on the surface index, the size and density of Au droplets show a clear distinction, observed throughout the temperature range. The results are systematically analyzed and discussed in terms of atomic force microscopy (AFM) images, cross-sectional line profiles, and Fourier filter transform (FFT) power spectra as well as the summary plots of the size and density.

## Background

**Figure 1:**
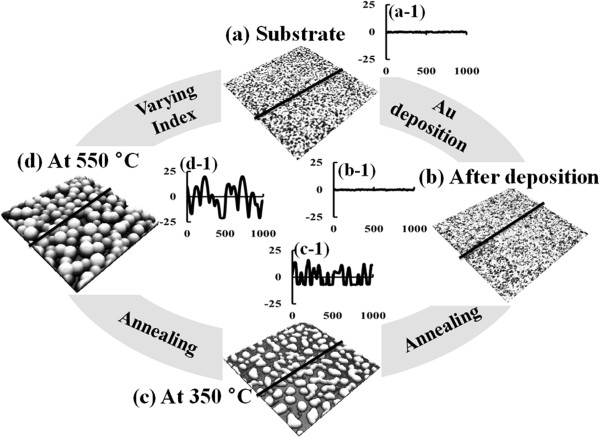
**Illustration of the fabrication process of self-assembled Au droplets on GaAs (111)A.** AFM side-view images of **(a)** the bare GaAs (111)A and **(b)** the surface after the 2.5-nm Au deposition. **(c)** Nucleation of wiggly Au nanostructure after annealing at 350°C. **(d)** Self-assembled Au droplets after annealing at 550°C. AFM side-view images of (a) to (d) are 1 × 1 μm^2^. The cross-sectional surface line profiles in (a-1) to (d-1) are acquired from the black lines in **(a)** to **(d)**.

Due to their increased cross-section and surface area as well as the size-dependent quantum confinement, semiconductor nanowires (NWs) have been successfully utilized in numerous device applications such as solar cells, LEDs, and FETs [1-8]. Until now, various semiconductor NWs have been successfully demonstrated through diverse epitaxial growth approaches including chemical vapor deposition [9,10], molecular beam epitaxy [11,12], and pulsed laser deposition [13,14]. Vapor–liquid-solid (VLS) [15-18] method has been widely adapted as a common growth mechanism in the forth-mentioned epitaxial approaches. The first successful fabrication of Si whisker on Si (111) was reported by Wagner et al., and they introduced a novel concept of growth approach called the ‘VLS’ growth [15]. Later, Morales et al. successfully demonstrated the fabrication of crystalline Si NWs by utilizing the VLS approach [16]. In the VLS growth, Au droplets serve as catalysts, and regardless of the materials and substrates utilized, the vapor-phase atoms could diffuse into the liquid-phase Au droplets [17,18]; from the supersaturated Au alloy droplets, the crystallization of NWs can occur at the liquid–solid interface due to the higher sticking probability at the interface [19-23]. In addition, the metallic nanoparticles were utilized in plasmonic applications such as solar cells and light emission enhancement [24-29]. The diameter, size, configuration, and even the density of NWs can innately be determined by those of the Au catalysts, and thus, the control of Au droplets is an essential step for the successful fabrication of the desired NWs. However, to date, the systematic studies on the evolution of Au droplets on various GaAs substrates are deficient, and therefore, in this paper, the detailed study on the evolution of the self-assembled Au droplets on GaAs (111)A, (110), (100), and (111)B is investigated. In order to investigate the detailed evolution process, feasible annealing temperatures were systematically tested ranging from 100°C to 550°C as briefly illustrated in Figure 1. Depending on the annealing temperature, the nucleation of self-assembled tiny Au clusters and wiggly Au nanostructures as shown in Figure 1c was clearly observed on various GaAs substrates. At increased annealing temperatures, the self-assembled Au droplets with fine uniformity were successfully fabricated on each GaAs index. The self-assembled Au droplets showed an opposite evolution trend of increased size including average height and lateral diameter with correspondingly decreased density as a function of annealing temperature, and the size and density evolution are systematically analyzed with the atomic force microscopy (AFM) images and cross-sectional line profiles as well as the summary plots. Under an identical growth condition, depending on the substrates utilized, the size and density of Au droplets show a clear disparity among various indices throughout the temperature range.

## Methods

In this study, the self-assembled Au droplets were fabricated on GaAs (111)A, (111)B, (110), and (100) representing the general zinc blende lattice indices in a pulsed laser deposition (PLD) system. To start with, various index samples were indium-bonded together on an Inconel holder side by side for uniformity per batch and then treated with a degassing process at 350°C for 30 min under 1 × 10^−4^ Torr. Subsequently, a total amount of 2.5 nm of Au was equally deposited on the samples at a rate of 0.5 Å/s and at an ionization current of 3 mA under 1 × 10^−1^ Torr in an ion coater chamber. With the aim of investigating the detailed evolution process of the self-assembled Au droplets, each growth was systematically carried out by varying the annealing temperatures (*T*_a_) at 100°C, 250°C, 300°C, 350°C, 400°C, 450°C, 500, and 550°C, respectively. For the systematic growths, the substrate temperature (*T*_s_) was ramped up to the target temperature at a ramp rate of 1.83°C/s under 1 × 10^−4^ Torr by a computer-operated recipe, and after reaching each target, a dwell time of 450 s was equally given to the samples. After the termination of each growth, the *T*_s_ was immediately quenched down to diminish the Ostwald ripening [30,31]. Following the fabrication, AFM was used for the characterization of surface morphologies, and XEI software was used for the data preparation and analysis of AFM top-view and side-view images and line profiles as well as the Fourier filter transform (FFT) power spectra. The FFT power spectrum represents the height information converted from the real spatial domain to the frequency domain, and thus, the horizontal (*x*) and vertical (*y*) information is converted by taking the reciprocal of the corresponding units of *x* and *y* from the AFM images; hence, the distribution of color patterns can present the distribution of frequent height with directionality.

## Results and discussion

**Figure 2:**
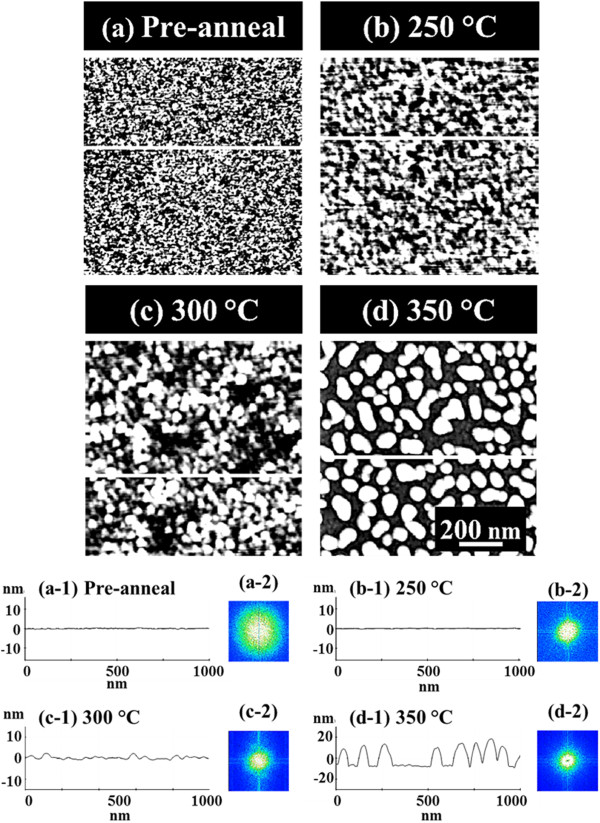
**Nucleation of self-assembled Au clusters and wiggling nanostructures.** The variation of annealing temperature (*T*_a_) done after 2.5-nm Au deposition on GaAs (111)A. The corresponding *T*_a_ is indicated with labels in the **(a-d)** AFM top-view images of 1 × 1 μm^2^. (a-1) to (d-1) are the cross-sectional surface line profiles acquired from the white lines in **(a)** to **(d)**. (a-2) to (d-2) show the corresponding 2-D FFT power spectra.

**Figure 3:**
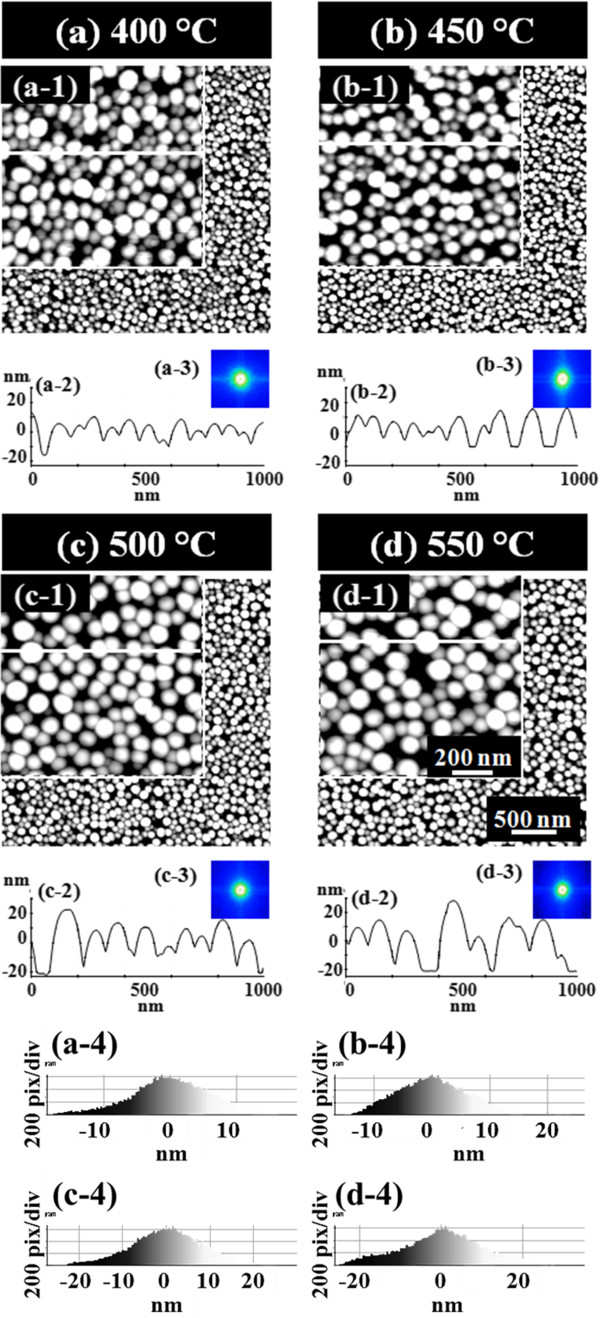
**Evolution of self-assembled Au droplets by increased *T***_**a **_**with 2.5-nm Au deposition on GaAs (111)A. (a-d)** AFM top-view images of 3 × 3 μm^2^ are shown with corresponding *T*_a_, and the enlarged images of 1 × 1 μm^2^ are shown in (a-1) to (d-1). (a-2) to (d-2) are cross-sectional surface line profiles acquired from the white lines in (a-1) to (d-1), and (a-3) to (d-3) show the 2-D FFT power spectra. Height distribution histograms are shown in (a-4) to (d-4).

Figure 2 presents the nucleation of the self-assembled Au clusters and the wiggling nanostructures induced by the variation of annealing temperature (*T*_a_) between 250°C and 350°C on GaAs (111)A. The AFM top-view images of 1 × 1 μm^2^ are presented in Figure 2a,b,c,d along with the cross-sectional line profiles in Figure 2 (a-1) to (d-1), acquired from the white lines in Figure 2a,b,c,d. The insets in Figure 2 (a-2) to (d-2) show the FFT power spectra. In general, on GaAs (111)A from a relatively flat surface morphology in Figure 2a,b, with the increased *T*_a_, the surface morphologies were drastically changed and gradually developed into Au clusters in Figure 3c and wiggly Au nanostructures in Figure 2d. In more detail, after the Au deposition before annealing, the surface showed a quite smooth topography as clearly observed by the AFM image in Figure 2a, and the line profile in Figure 2 (a-1) and the corresponding FFT spectrum in Figure 2 (a-2) showed a quite broad round pattern due to the narrow random surface modulation. At the *T*_a_ of 250°C, the diffusion of Au adatoms was induced as shown in Figure 2b, but the surface modulation was only slightly increased as evidenced by the line profile in Figure 2 (b-1). The FFT spectrum in Figure 2 (b-2) became smaller with a round pattern. With the increased thermal energy at 300°C, the diffusion of adatoms was further enhanced, and as a result, there was nucleation of tiny Au clusters with a slightly bumpy morphology as shown in Figure 2c and (c-1). Finally, at the *T*_a_ of 350°C, as clearly seen with the AFM image in Figure 2d and the line profile in Figure 2 (d-2), a sharp transition from the Au clusters to the wiggly nanostructures occurred with a height modulation of approximately ±10 nm as clearly evidenced by the line profiles of Figure 2 (c-1) and (d-1). The FFT pattern size was further reduced with the increased height modulation and became a symmetric circle as there was no apparent directionality of Au nanostructures. The Au clusters and wiggly nanostructures can be formed based on the Volmer-Weber growth mode [32,33]. Given that the bonding energy among Au adatoms (*E*_a_) is greater than that between Au adatoms and GaAs surface atoms (*E*_i_), Au adatoms can be merged together to nucleate the Au clusters at a relatively lower *T*_a_, and the wiggly Au nanostructures can result at an increased *T*_a_. This transition of surface morphology associated with the nucleation of the Au clusters and wiggly nanostructures appears to be unique to GaAs. For example, on Si (111) neither this type of transition nor the Au clusters or the wiggly Au nanostructures were observed during the evolution of the self-assembled Au droplets while varying the *T*_a_ between 50°C and 850°C [34], but very high density dome-shaped Au droplets were observed throughout the temperature range. In short, with the increased *T*_a_ on GaAs (111)A, apparent transitions of surface morphologies at each *T*_a_ were clearly observed and the height modulation was gradually enlarged as a function of *T*_a_; a sharp transition was observed at 350°C with a surface modulation of approximately ±10 nm due to the increased diffusion of Au adatoms induced by the enhanced thermal energy.

**Figure 4:**
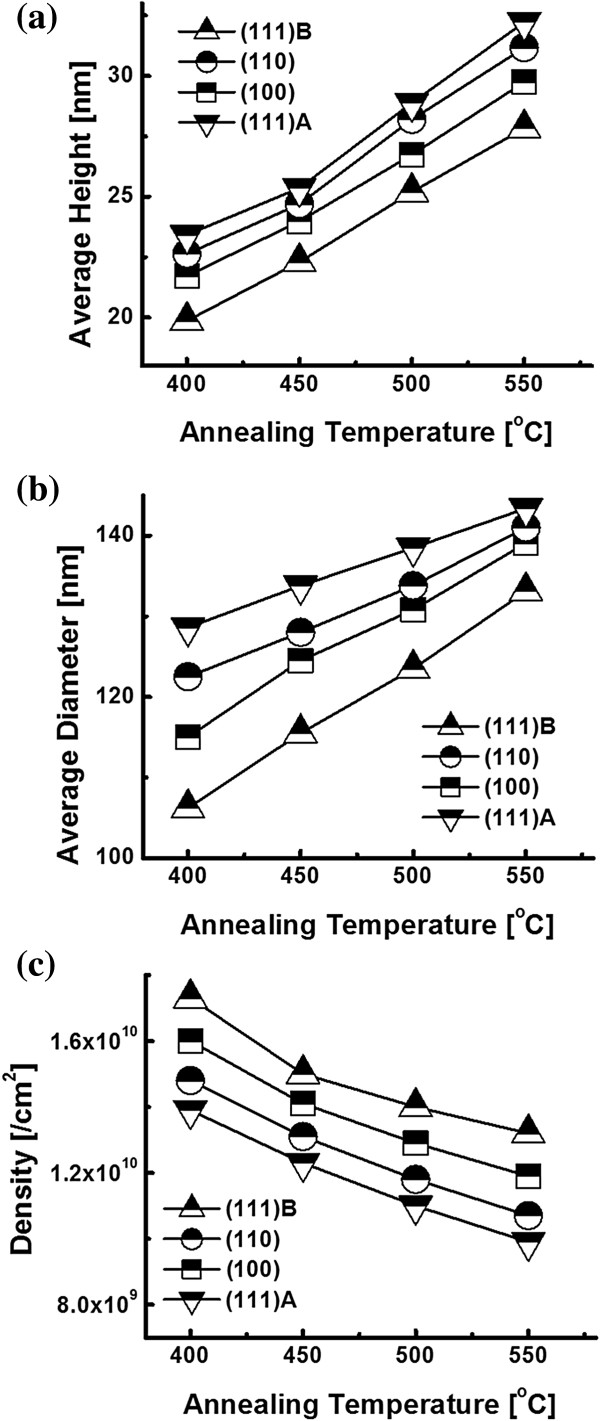
**Summary plots.** Plots of the **(a)** average height, **(b)** average lateral diameter, and **(c)** average density of self-assembled Au droplets on various GaAs surfaces at the corresponding annealing temperature between 400°C and 550°C.

Figure 3 shows the evolution of self-assembled Au droplets with further increased *T*_a_ between 400°C and 550°C on GaAs (111)A. AFM top-view images in Figure 3a,b,c,d show the large areas of 3 × 3 μm^2^, and the insets of Figure 3 (a-1) to (d-1) are the enlarged areas of 1 × 1 μm^2^. The surface line profiles in Figure 3 (a-2) to (d-2), the FFT power spectra in Figure 3 (a-3) to (d-3), and the height distribution histograms (HDHs) in Figure 3 (a-4) to (d-4) are respectively presented. Figure 4 shows the summary plots of the average height (AH) in Figure 4a, the lateral diameter (LD) in Figure 4b, and the average density (AD) in Figure 4c of the self-assembled Au droplets at each *T*_a_ on various GaAs substrates. Table 1 summarizes the corresponding values. In general, between 400°C and 550°C, the self-assembled dome-shaped Au droplets were successfully fabricated as shown in Figure 3. Due to the enhanced diffusion of Au adatoms at increased thermal energy, given *E*_a_ > *E*_i_, the wiggly Au nanostructures preferentially evolve into the dome-shaped Au droplets to minimize the surface energy [35]. In terms of the size and density evolution, as clearly shown in Figure 4a,b,c, the size including the AH and LD of the Au droplets was gradually increased, while the density was correspondingly decreased as a function of the *T*_a_ on GaAs (111)A. In more detail, at an increased *T*_a_ of 400°C, finally, the self-assembled Au droplets were fabricated and we can clearly observe the apparent transition from the wiggly Au nanostructures at 350°C to the dome-shaped Au droplets at 400°C. The AH was 23.4 nm, the LD was 128.6 nm, and the AD was 1.39 × 10^10^ cm^−2^ as shown in Table 1. The HDH was approximately ±15 nm as shown in Figure 3 (a-4). At 450°C, the Au droplets grew larger in size and showed a lower density as shown in Figure 4. The AH was increased by × 1.09 and became 25.4 nm, and the LD was increased by × 1.04 and became 133.8 nm as shown in Table 1. The density was dropped by × 1.13 and became 1.23 × 10^10^ cm^−2^. Likewise, at 500°C, the size of the Au droplets was further increased, and the density was correspondingly decreased as shown in Figure 3c. The AH and LD were increased by × 1.14 and × 1.04 and became 28.9 and 138.5 nm, respectively, while the AD was decreased by × 1.04 and became 1.23 × 10^10^ cm^−2^. The HDH was now further extended with the increased size to over ±20 nm as shown in Figure 3 (c-4). Finally, with the 550°C annealing temperature, the Au droplets continually grew in size and the density was constantly decreased as shown in Figure 4. The AH and LD were increased by × 1.11 and × 1.04 and became 32.2 and 143.4 nm. Likewise, the AD was down by × 1.11 and became 9.9 × 10^9^ cm^−2^ as shown in Table 1. The HDH in Figure 3 (d-4) now became clearly over ±20 nm wide along with the increased height of Au droplets. The self-assembled Au droplets on GaAs (111)A with the *T*_a_ variation between 400°C and 550°C showed quite excellent uniformity as witnessed in the symmetric round FFT power spectra of Figure 3 (a-3) to (d-3) and showed an overall increased size with decreased density as a function of the *T*_a_. The size and density evolution induced by the variation of the *T*_a_ can be simply explained with the following equation [36]. The diffusion length (*l*_D_) can be expressed as $l_{D}=\sqrt{\mathrm{D\tau}}$ where *D* is the surface diffusion coefficient and *τ* is the residence time of atoms. *D* can be written as  *D* ∝ *T*_sub_ where *T*_sub_ is the substrate temperature, namely *T*_a_ in this case. With the increased *T*_a_, the *D* proportionally increases and it results in an increased *l*_D_. With the increased *l*_D_, the density of the Au droplets can be decreased, given the stronger bonding energy between Au atoms (*E*_a_ > *E*_i_). In this thermodynamic equilibrium system, in order to keep the energy of the whole system in the lowest state, bigger droplets tend to absorb nearby adatoms to lower the surface energy, and thus, the size can grow larger and the density can be reduced until reaching the equilibrium. Thus, this type of size and density evolution was witnessed in Ga and In metal droplets [35,37,38] and nanostructures [39-41] on various semiconductor substrates.

**Table 1:** Summary of AH, LD, and AD of self-assembled Au droplets

	**I**	***T***_**a **_**(°C)**			
		**400**	**450**	**500**	**550**
Average height (AH) [nm]	(111)A	23.4	25.4	28.9	32.2
	(110)	22.6	24.7	28.2	31.2
	(100)	21.7	24.0	26.7	29.7
	(111)B	19.9	22.3	25.2	27.8
Average lateral diameter (LD) [nm]	(111)A	128.6	133.8	138.5	143.4
	(110)	122.5	128	133.8	141
	(100)	115	124.5	130.8	139.1
	(111)B	106.2	115.5	123.5	133.1
Average density (AD) [×10^8^ cm^−2^]	(111)A	139	123	110	99
	(110)	148	131	118	107
	(100)	160	141	129	119
	(111)B	173	150	140	132

The Au droplets were fabricated by annealing between 400°C and 550°C on GaAs (111)A, (110), (100), and (111)B. I, index of substrates; *T*_a_, annealing temperature.

**Figure 5:**
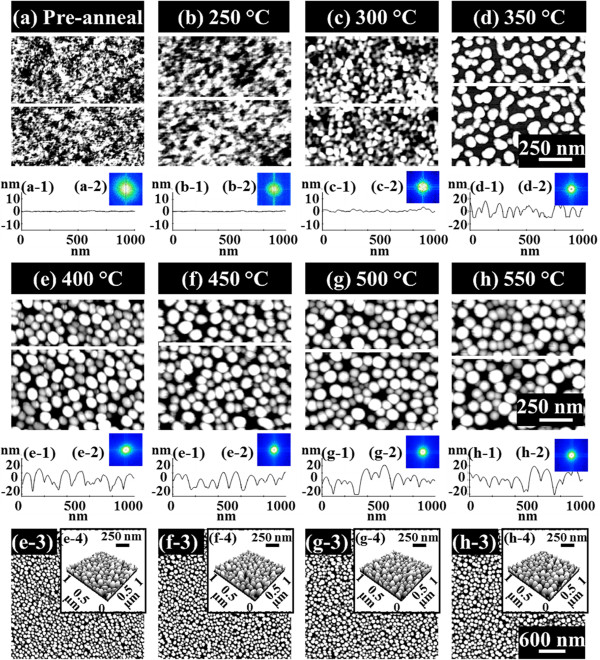
**Summary of the evolution process on GaAs (110).** Evolution of self-assembled Au droplets on GaAs (110) by the variation of *T*_a_ between 250°C and 550°C for 450 s with 2.5-nm Au deposition. Results are presented with **(a-h)** the AFM top-view images of 1 × 1 μm^2^, the corresponding surface cross-sectional line profiles in (a-1) to (h-1), and the FFT power spectra in (a-2) to (h-2). Larger scale AFM top-view images of 3 × 3 μm^2^ are presented in (e-3) to (h-3), and the AFM side-view images of 3 × 3 μm^2^ are shown in (e-4) to (h-4).

**Figure 6:**
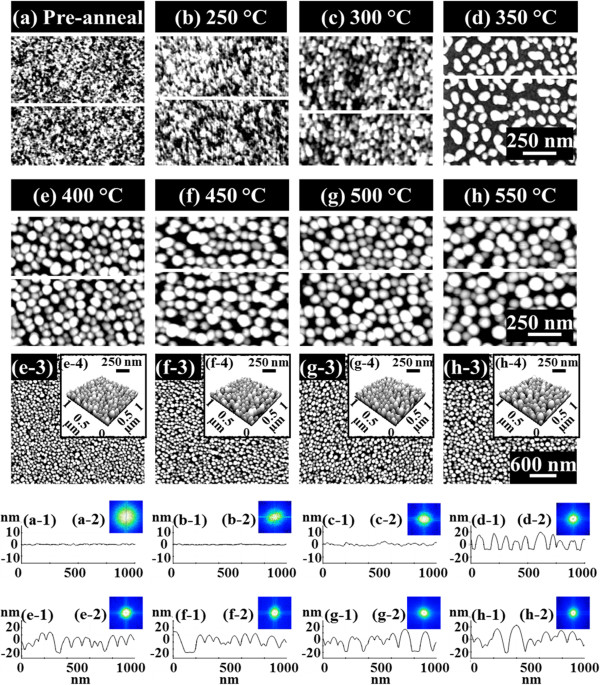
**Temperature effect on the evolution of self-assembled Au droplets on GaAs (100).** Au droplets were fabricated by annealing between 250°C and 550°C for 450 s with 2.5-nm Au deposition. The evolution process is presented with **(a-h)** the AFM top-view images of 1 × 1 μm^2^ and the line profiles in (a-1) to (h-1) with the corresponding FFT power spectra in (a-2) to (h-2). AFM top-view images of 3 × 3 μm^2^ are shown in (e-3) to (h-3), and the insets of AFM side-view images of 1 × 1 μm^2^ are shown in (e-4) to (h-4).

**Figure 7:**
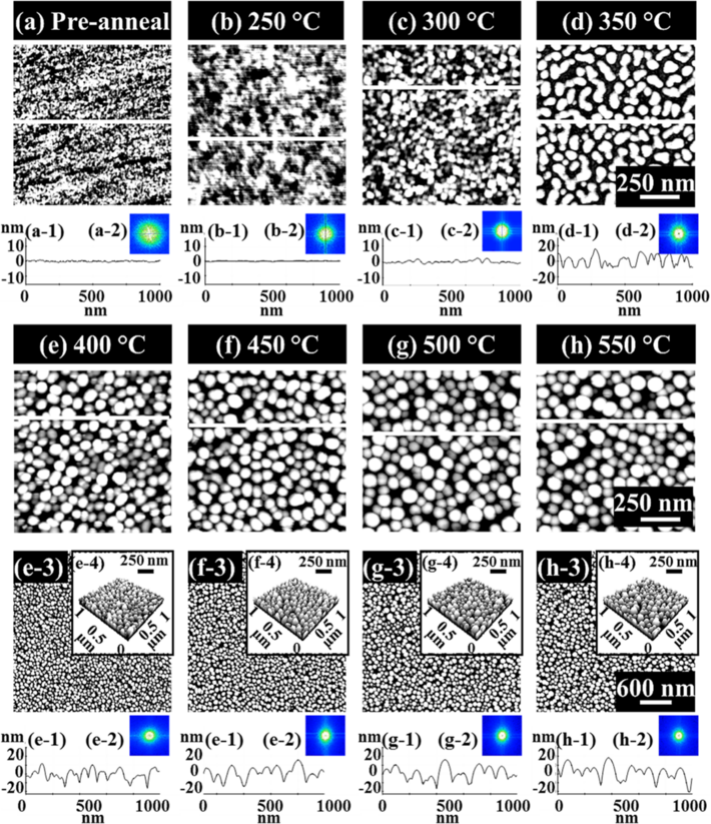
**The evolution of self-assembled Au droplets on GaAs (111)B.** The results are shown with the **(a-h)** AFM top-view images of 1 × 1 μm^2^ and the corresponding cross-sectional line profiles in (a-1) to (h-1) with the FFT power spectra in (a-2) to (h-2). The AFM top-view images of 3 × 3 μm^2^ are presented in (e-3) to (h-3), and the AFM side-view images of 1 × 1 μm^2^ are shown in (e-4) to (h-4) along with the corresponding height distribution histograms in (e-5) to (h-5).

Figure 5 summarizes the evolution process of the self-assembled Au droplets on GaAs (110) induced by the variation of the *T*_a_ between 250°C and 550°C, and similarly, Figures 6 and 7 show that on GaAs (100) and (111)B. In general, the evolution process of the Au droplets on GaAs (110), (100), and (111)B showed quite a similar behavior to that on GaAs (111)A in terms of the nucleation of Au clusters and wiggly nanostructures as shown in Figure 2 and the evolution of size and density as a function of the *T*_*a*_ as summarized with the plots in Figure 4. For example, on GaAs (110) between 250°C and 350°C, the nucleation of Au clusters and wiggly Au nanostructures was clearly observed as shown in Figure 5b,c,d, and between 400°C and 550°C, the self-assembled dome-shaped Au droplets were successfully fabricated as shown in Figure 5e,f,g,h. The size of droplets on GaAs (110) was also constantly increased as a function the *T*_a_, while the density was correspondingly decreased as clearly shown in Figure 4. However, the size of Au droplets on GaAs (110) was slightly smaller than that on GaAa (111)A, putting the (110) line below the (111)A in Figure 4a,b, and as a result, based on the thermodynamic description, the density was slightly higher throughout the whole temperature range, marking the (110) line above the (111)A in Figure 4c. For example, at 400°C, the AH, LD, and AD were 22.6 nm, 122.5 nm, and 1.48 × 10^10^ cm^−2^, which are 3.42% and 4.47% smaller in size and 6.47% higher in density as compared to those on GaAs (111)A. Similarly, at 550°C, the size and density of droplets on (110) were 31.2 nm (AH), 141 nm (LD), and 1.07 × 10^10^ cm^−2^ (AD), which are 3.11% smaller in AH and 1.67% smaller in LD and 8.08% higher in AD. In short, the self-assembled Au droplets on GaAs (110) clearly showed smaller size and correspondingly higher density as compared to those on GaAs (111)A throughout the *T*_a_ range. In the meantime, on GaAs (100) and (111)B, the nucleation of Au clusters and wiggly nanostructures was also clearly observed between 250°C and 350°C as shown in Figures 6b,c,d and 7b,c,d, and the self-assembled Au droplets were also successfully fabricated between 400°C and 550°C as shown in Figure 6e,f,g,h and 7e,f,g,h. In the same way, on both GaAs (100) and (111)B, the size of the Au droplets was constantly increased as a function of *T*_a_ and the density was correspondingly decreased. Depending on the surface index, there appeared a clear difference in size and density between the indices, and this trend constantly appeared throughout the *T*_a_ range as clearly shown in Figure 4. For instance, GaAs (111)B showed the smallest Au droplets at each point of the *T*_a_, putting the (111)B line at the bottom of the plots (a) and (b), and the (100) was the second. Then, the (110) showed further increased size, and finally, the biggest droplets were fabricated on GaAs (111)A. In terms of the density, GaAs (111)B showed the highest at each point of the *T*_a_, followed by (100), (110), and (111)A. The Miller index [110] of zinc blende lattice is located at 45° toward [010] from the [100], and these two indices with [111] can represent the general zinc blende indices except for the high index. As discussed, the diffusion length (*l*_D_) can be directly related to the *T*_a_ and thus can affect the size and density of Au droplets. The *l*_D_ can also be related to the root mean squared (RMS) surface roughness (*R*_q_) caused by several factors such as the dangling bond density, atomic step density, and surface reconstruction [42-46]. If the *R*_q_ value of one surface is relatively lower, the surface would possess longer *l*_D_, and it can result in a larger size and a lower density of Au droplets. The measurements of *R*_q_ values on the GaAs indices are as follows: (111)A, 0.289 nm; (110), 0.305 nm; (100), 0.322 nm; and (111)B, 0.291 nm. GaAs (111)A showed the lowest *R*_q_, and (110) had a slightly increased value; thus, this can explain the larger size and the lower density of droplets on GaAs (111)A as shown in Figure 4. Similarly, we can relate the decreased size and the increased density of Au droplets on GaAs (100) as compared to those on (110) with the increased *R*_q_. However, the (111)B surface showed similar *R*_q_ to the (111)A, and the results nevertheless showed the smallest size with the highest density. The type-A GaAs surface is characterized to be Ga-rich, while the type-B surface is As-rich [42]. The Ga-rich surface can possess a higher interface energy than the As-rich surface based on the atomistic modeling of the Au droplet-GaAs interface [47], and thus, the reduced diffusion of Au atoms on type-B surface can lead to a lower *l*_D_; hence, the smaller size of droplets with a higher density can result. In short, on various GaAs surfaces, the evolution process of the self-assembled Au droplets was clearly demonstrated, and they showed quite similar behaviors in terms of the size and density evolution while keeping the difference between indices throughout the whole *T*_a_ range.

## Conclusions

The evolution of the self-assembled Au droplets has been successfully demonstrated on GaAs (111)A, (110), (100), and (111)B through the variation of annealing temperature throughout the feasible annealing temperature (*T*_a_) range between 250°C to 550°C. The resulting Au nanostructures were systematically analyzed in terms of AFM images, cross-sectional line profiles, height distribution histograms, and FFT power spectra. The unique nucleation stages of the Au clusters and wiggly nanostructures were observed on various GaAs surfaces at the *T*_a_ range between 250°C and 350°C, and the self-assembled dome-shaped Au droplets with excellent uniformity were successfully fabricated between 400°C and 550°C. The average height and lateral diameter of the Au droplets were gradually increased with the increased *T*_a_, and the average density was correspondingly decreased at each *T*_a_ point. The nucleation and the formation of Au droplets were described based on the Volmer-Weber growth mode, namely *E*_a_ > *E*_i_. The evolution of the size and density of Au droplets was described in terms of the *l*_D_ of Au adatoms in relation with the thermal dynamic equilibrium along with the *T*_a_. In addition, an apparent distinction in the size and density of Au droplets between various GaAs indices was clearly observed, and it was maintained throughout the *T*_a_ range GaAs (111)A > (110) > (100) > (111)B in size and vice versa in diameter, and the trend was described in relation between the *R*_q_ and *l*_D_. This study can find applications in the nanowire fabrications on various GaAs surfaces.

## Competing interests

The authors declare that they have no competing interests.

## Authors' contributions

ML, MS, and JL participated in the experiment design and carried out the experiments. ML, MS, EK, and JL participated in the analysis of data. ML, MS, and JL designed the experiments and testing methods. ML and JL carried out the writing. All authors helped in drafting and read and approved the final manuscript.

## References

[B1] Steffen B Carsten P€u Timur F Oliver B Grahn HT Lutz G Henning R Suitability of Au- and self-assisted GaAs nanowires for optoelectronic applications Nano Lett 2011 9 1276 1279 10.1021/nl104316t 21319838

[B2] Wen C-Y Reuter MC Bruley J Tersoff J Kodambaka S Stach EA Ross FM Formation of compositionally abrupt axial heterojunctions in silicon-germanium nanowires Science 2009 9 1247 1250 10.1126/science.1178606 19965471

[B3] Mahpeykar SM Koohsorkhi J Ghafoori-fard H Ultra-fast microwave-assisted hydrothermal synthesis of long vertically aligned ZnO nanowires for dye-sensitized solar cell application Nanotechnology 2012 9 165602(1) 165602(7) 22460691 10.1088/0957-4484/23/16/165602

[B4] Haofeng L Rui J Chen C Zhao X Wuchang D Yanlong M Deqi W Xinyu L Tianchun Y Influence of nanowires length on performance of crystalline silicon solar cell Appl Phys Lett 2011 9 151116(1) 151116(3)

[B5] Tae Hoon S Bo Kyoung K GangU S Changhyup L Myung Jong K Hyunsoo K Eun-Kyung S Graphene-silver nanowire hybrid structure as a transparent and current spreading electrode in ultraviolet light emitting diodes Appl Phys Lett 2013 9 051105(1) 051105(5)

[B6] Shirak O Shtempluck O Kotchtakov V Bahir G Yaish YE High performance horizontal gate-all-around silicon nanowire field-effect transistors Nanotechnology 2012 9 395202(1) 395202(8) 22971804 10.1088/0957-4484/23/39/395202

[B7] Jae-Hyuk A Sung-Jin C Jin-Woo H Tae Jung P Sang Yup L Yang-Kyu C Double-gate nanowire field effect transistor for a biosensor Nano Lett 2010 9 2934 2938 10.1021/nl1010965 20698606

[B8] Frajtag P Hosalli AM Bradshaw GK Nepal N El-Masry NA Bedair SM Improved light-emitting diode performance by conformal overgrowth of multiple quantum wells and fully coalesced p-type GaN on GaN nanowires Appl Phys Lett 2011 9 143104(1) 143104(3)

[B9] Ying X Linyou C Sonia C-B Sonia E Jordi A Francesca Peiro MH Zardo I Morante JR Brongersma ML Morral AF single crystalline and core–shell indium-catalyzed germanium nanowires—a systematic thermal CVD growth study Nanotechnology 2009 9 245608(1) 245608(9) 10.1088/0957-4484/20/24/245608 19471084

[B10] Jorg KNL DjamilaBahloul H Daniel K Michael W Thierry M Bernd S TEM characterization of Si nanowires grown by CVD on Si pre-structured by nanosphere lithography Mater Sci Semicond Process 2008 9 169 174 10.1016/j.mssp.2008.09.016

[B11] Cai Y Wong TL Chan SK Sou IK Su DS Wang N Growth behaviors of ultrathin ZnSe nanowires by Au-catalyzed molecular-beam epitaxyAppl Phys Lett 2008 9 233107(1) 233107(3)

[B12] Tchernycheva M Harmand JC Patriarche G Travers L Cirlin GE Temperature conditions for GaAs nanowire formation by Au-assisted molecular beam epitaxy Nanotechnology 2006 9 4025 4030 10.1088/0957-4484/17/16/005 21727532

[B13] Kazuki N Takeshi Y Hidekazu T Tomoji K Epitaxial growth of MgO nanowires by pulsed laser deposition Appl Phys Lett 2007 9 124304(1) 124304(4)

[B14] Bjorn E Vladimir S Andreas B Silke C Growth of axial SiGe heterostructures in nanowires using pulsed laser deposition Nanotechnology 2011 9 305604(1) 305604(8) 10.1088/0957-4484/22/30/305604 21705828

[B15] Wagner RS Ellis WC Vapor liquid solid mechanism of single crystal growth Appl Phys Lett 1964 9 89 90 10.1063/1.1753975

[B16] Morales AM Lieber CM Laser ablation method for the synthesis of crystalline semiconductor nanowires Science 1998 9 208 208 10.1126/science.279.5348.208 9422689

[B17] Volker S Ulrich G How nanowires grow Science 2007 9 698 698 10.1126/science.1142951 17478707

[B18] Khac An D Khang Dao D Dai Nguyen T Tuan Phan A Hung Manh D The effects of Au surface diffusion to formation of Au droplets/clusters and nanowire growth on GaAs substrate using VLS method Mater Electron 2012 9 2065 2074 10.1007/s10854-012-0704-y

[B19] Borgstrom M Deppert K Samuelson L Seifert W Size- and shape-controlled GaAs nano-whiskers grown by MOVPE: a growth study J Cryst Growth 2004 9 18 22 10.1016/j.jcrysgro.2003.08.009

[B20] Yi C Lauhon LJ Gudiksen MS Jianfang W Lieber CM Diameter-controlled synthesis of single-crystal silicon nanowires Appl Phys Lett 2001 9 2214 2216 10.1063/1.1363692

[B21] Pin Ann L Dong L Samantha R Xuan P Gao A Mohan Sankaran R Shape-controlled Au particles for InAs nanowire growth Nano Lett 2012 9 315 320 10.1021/nl2036035 22142439

[B22] Hannon JB Kodambaka S Ross FM Tromp RM The influence of the surface migration of gold on the growth of silicon nanowires Nature 2006 9 69 71 10.1038/nature04574 16452928

[B23] Jianwei Z Lirong Q Yong Z Yonghao H Qing G Lide Z Catalytic growth of cubic phase ZnO nanowires with jagged surface Micro Nano Lett 2010 9 336 339 10.1049/mnl.2010.0126

[B24] Jiang W Seungyong L Reddy VR Manasreh MO Weaver BD Yakes MK Furrow CS Kunets VP Benamara M Salamo GJ Photoluminescence plasmonic enhancement in InAs quantum dots coupled to gold nanoparticle Mater Lett 2011 9 3605 3608 10.1016/j.matlet.2011.08.019

[B25] Guang Z Fengfang S Tian L Likun P Zhuo S Au nanoparticles as interfacial layer for CdS quantum dot-sensitized solar cells Nanoscale Res Lett 2010 9 1749 1754 10.1007/s11671-010-9705-z 21124643 PMC2964506

[B26] Catchpole KR Polman A Design principles for particle plasmon enhanced solar cells Appl Phys Lett 2008 9 191113(1) 191113(3)

[B27] Jiang W Mangham SC Reddy VR Manasreh MO Weaver BD Surface plasmon enhanced intermediate band based quantum dots solar cell Sol Energy Mater Sol Cells 2012 9 44 49

[B28] Zhang YF Wang YF Chen N Wang YY Zhang YZ Zhou ZH Wei LM Photovoltaic enhancement of Si solar cells by assembled carbon nanotubes Nano-Micro Lett 2010 9 22 25

[B29] Jiunn-Woei L Huang-Chih C Mao-Kuen K Plasmonic Fano resonance and dip of Au-SiO_2_-Au nanomatryoshka Nanoscale Res Lett 2013 9 468(1) 486(8) 10.1186/1556-276X-8-468 PMC3829662 24206789

[B30] Jian Hua Y Elder KR Hong G Martin G Theory and simulation of Ostwald ripening Phys Rev B 1993 9 14110 14125 10.1103/PhysRevB.47.14110 10005752

[B31] Alloyeau D Oikawa T Nelayah J Wang G Ricolleau C Following Ostwald ripening in nanoalloys by high-resolution imaging with single-atom chemical sensitivity Appl Phys Lett 2012 9 121920(1) 121920(3)

[B32] Zhenyu Z Lagally MG Atomistic processes in the early stages of thin-film growth Science 1997 9 377 383 10.1126/science.276.5311.377 9103189

[B33] Abraham DB Newman CM Equilibrium Stranski-Krastanow and Volmer-Weber models Europhysics Lett 2009 9 16002(p1) 16002(p4)

[B34] Sui M Li MY Kim ES Lee JH Annealing temperature effect on self-assembled Au droplets on Si (111) Nanoscale Res Lett 2013 9 525 10.1186/1556-276X-8-525 24330583 PMC3878746

[B35] Lei G Yusuke H Ming-Yu L Jiang W Sangmin S Sang-Mo K Eun-Soo K Wang ZM Jihoon L Salamo GJ Observation of Ga metal droplet formation on photolithographically patterned GaAs (100) surface by droplet epitaxy IEEE Trans Nanotechnol 2012 9 985 991

[B36] Rijnders G Blank DHA Pulsed Laser Deposition of Thin Films: Applications-Led Growth of Functional Materials, Chapter 8 2007 USA: Wiley-Interscience, USA 179 180

[B37] Jihoon L Zhiming W Yusuke H Eun-Soo K Namyoung K Seunghyun P Cong W Salamo GJ Various configurations of In nanostructures on GaAs (100) by droplet epitaxy Cryst Eng Comm 2010 9 3404 3408 10.1039/c0ce00057d

[B38] Ziad Y Abu W Wang ZM Lee JH Salamo GJ Optical behavior of GaAs/AlGaAs ring-like nanostructures Nanotechnology 2006 9 4037 4040 10.1088/0957-4484/17/16/007 21727534

[B39] Lee JH Wang ZM Black WT Kunets VP Mazur YI Salamo GJ Spatially localized formation of InAs quantum dots on shallow mesa- and trench patterns regardless of crystallographic directions Adv Funct Mater 2007 9 3187 10.1002/adfm.200700066

[B40] Lee JH Wang ZM Kim ES Kim NY Park SH Salamo GJ Self-assembled InGaAs tandem nanostructures consisting a hole and pyramid on GaAs (311)A by droplet epitaxy Phys Status Solidi (a) 2010 9 348 10.1002/pssa.200925406

[B41] Lee JH Sablon K Wang ZM Salamo GJ Evolution of InGaAs quantum dot molecules J Appl Phys 2008 9 054301 10.1063/1.2890149

[B42] Wang ZM Seydmohamadi S Lee JH Salamo GJ Surface ordering of (In, Ga)As quantum dots controlled by GaAs substrate indexes Appl Phys Lett 2004 9 5031 10.1063/1.1823590

[B43] Biegelsen DK Bringans RD Northrup JE L E Surface reconstructions of GaAs(100) observed by scanning tunneling microscopy Phys ReV B 1990 9 5701 5711 10.1103/PhysRevB.41.5701 9994453

[B44] Laukkanen P Kuzmin M Perälä RE Ahola M Mattila S Väyrynen I Electronic and structural properties of GaAs(100) (2 × 4) and InAs(100) (2 × 4) surfaces studied by core-level photoemission and scanning tunneling microscopy J Phys ReV B 2005 9 045321

[B45] Jiang W Wang ZM Li AZ Shibin L Salamo GJ Surface mediated control of droplet density and morphology on GaAs and AlAs surfaces Phys Status Solidi (RRL)-Rapid Res Lett 2010 9 371 373 10.1002/pssr.201004402

[B46] Duke CB Mailhiot C Paton A Kahn A Stiles K Shape and growth of InAs quantum dots on high-index GaAs(113)A, B and GaAs(2 5 11)A, B substrates J Vac Sci Technol A 1986 9 947 952 10.1116/1.573762

[B47] Sakong S Du YA Kratzer P Atomistic modeling of the Au droplet–GaAs interface for size-selective nanowire growth Phys ReV B 2013 9 155309

